# TRANSITIONS TO LIVING ALONE AND PSYCHOLOGICAL WELL-BEING OF OLDER ADULTS IN THE U.S: ARE THERE ANY RACE DISPARITIES?

**DOI:** 10.1093/geroni/igad104.3672

**Published:** 2023-12-21

**Authors:** Wuyi Dong, Qian Song

**Affiliations:** University of Massachusetts Boston, Boston, Massachusetts, United States; University of Massachusetts Boston, Boston, Massachusetts, United States

## Abstract

We extend the existing literature on living alone and psychological well-being in older adults by focusing on the transition process from other living arrangements to living alone. We examine how transitioning to living alone is associated with psychological well-being scores (measured by 21-item Ryff Measures of Psychological Well-being) and how the associations vary across racial and ethnic groups. Using data from 2016 and 2020 Health and Retirement Study, we specify the transitions to living alone from four living arrangements: living with spouse/partner only, living with spouse/partner and older parents, living with a spouse/partner, children, and parents, and living with others. Respondents consistently living alone were the reference group. Our analysis involves adults aged 50 and above (n=3160). After controlling for final psychological well-being scores and adjusting for age, gender, marital status, and education in 2016, results from multiple linear regression models suggest that the transitions from living with spouse/partner only to living alone are associated with significantly lower scores of psychological well-being (β= -1.71, p<.01) than those consistently living alone. However, no statistically significant differences were observed between individuals transitioning from the other three living arrangements to living alone and those consistently living alone. The moderation effect shows that older Blacks experienced increased psychological well-being when transitioning from living with a spouse/partner to living alone (β=2.26) while their White counterparts had decreased psychological well-being during the same transition. This underscores the significance of the transition experience and emphasizes race disparities in its association with psychological health outcomes.

